# Microwave-Induced Chemotoxicity of Polydopamine-Coated Magnetic Nanocubes

**DOI:** 10.3390/ijms160818283

**Published:** 2015-08-06

**Authors:** Khachatur Julfakyan, Yevhen Fatieiev, Shahad Alsaiari, Lin Deng, Alaa Ezzeddine, Dingyuan Zhang, Vincent M. Rotello, Niveen M. Khashab

**Affiliations:** 1 Smart Hybrid Materials (SHMs) Laboratory, Advanced Membranes and Porous Materials Center, King Abdullah University of Science and Technology (KAUST), Thuwal 23955-6900, Saudi Arabia; E-Mails: khachatur.julfakyan@kaust.edu.sa (K.J.); yevhen.fatieiev@kaust.edu.sa (Y.F.); shahad.alsaiari@kaust.edu.sa (S.A.); lin.deng@kaust.edu.sa (L.D.); alaa.ezzeddine@kaust.edu.sa (A.E.); zhang-chem@hotmail.com (D.Z.); 2 Key Laboratory of Synthetic and Self-Assembly Chemistry for Organic Functional Molecules, Shanghai Institute of Organic Chemistry, Chinese Academy of Sciences, Shanghai 200032, China; 3 Department of Chemistry, University of Massachusetts Amherst, Amherst, MA 01003, USA; E-Mail: rotello@chem.umass.edu

**Keywords:** FeCo nanocubes, polydopamine, microwave radiation, nanomedicine

## Abstract

Polydopamine-coated FeCo nanocubes (PDFCs) were successfully synthesized and tested under microwave irradiation of 2.45 GHz frequency and 0.86 W/cm^2^ power. These particles were found to be non-toxic in the absence of irradiation, but gained significant toxicity upon irradiation. Interestingly, no increase in relative heating rate was observed when the PDFCs were irradiated in solution, eliminating nanoparticle (NP)-induced thermal ablation as the source of toxicity. Based on these studies, we propose that microwave-induced redox processes generate the observed toxicity.

## 1. Introduction

Microwave ablation has been clinically evaluated for use in the treatment of metastatic and early stage breast cancer, hepatocellular carcinoma, and lung cancer [1,2,3,4,5]. Focused microwave irradiation over a desired area leads to local application of heating to induce irreversible cell injury. However, indirect heating and killing of healthy tissues remains one of the major drawbacks of microwave therapy, as well as magnetic hyperthermia and near-infrared photothermal therapy [6,7].

Developing new nanomaterials that can be triggered by external stimuli without extreme temperature elevation can provide a safer route to utilize radiation techniques for cancer therapy. Recently core shell nanocubes with exceptional specific absorption rate have been reported by Cheon *et al.*, demonstrating the influence of cubic shape on magnetic properties [8]. These FeCo nanocubes feature defined shape, ease of surface functionalization, and high magnetization [9,10,11]. Herein, we report the fabrication of polydopamine coated [12,13,14,15,16] FeCo nanocubes with low inherent toxicity. Microwave irradiation of 2.45 GHz frequencies (similar to Bluetooth and microwave ovens) provided significantly enhanced toxicity. To the best of our knowledge, this represents the first study of low energy microwave radiation on such systems.

## 2. Results and Discussion

### 2.1. Synthesis and Characterization

**Figure 1:**
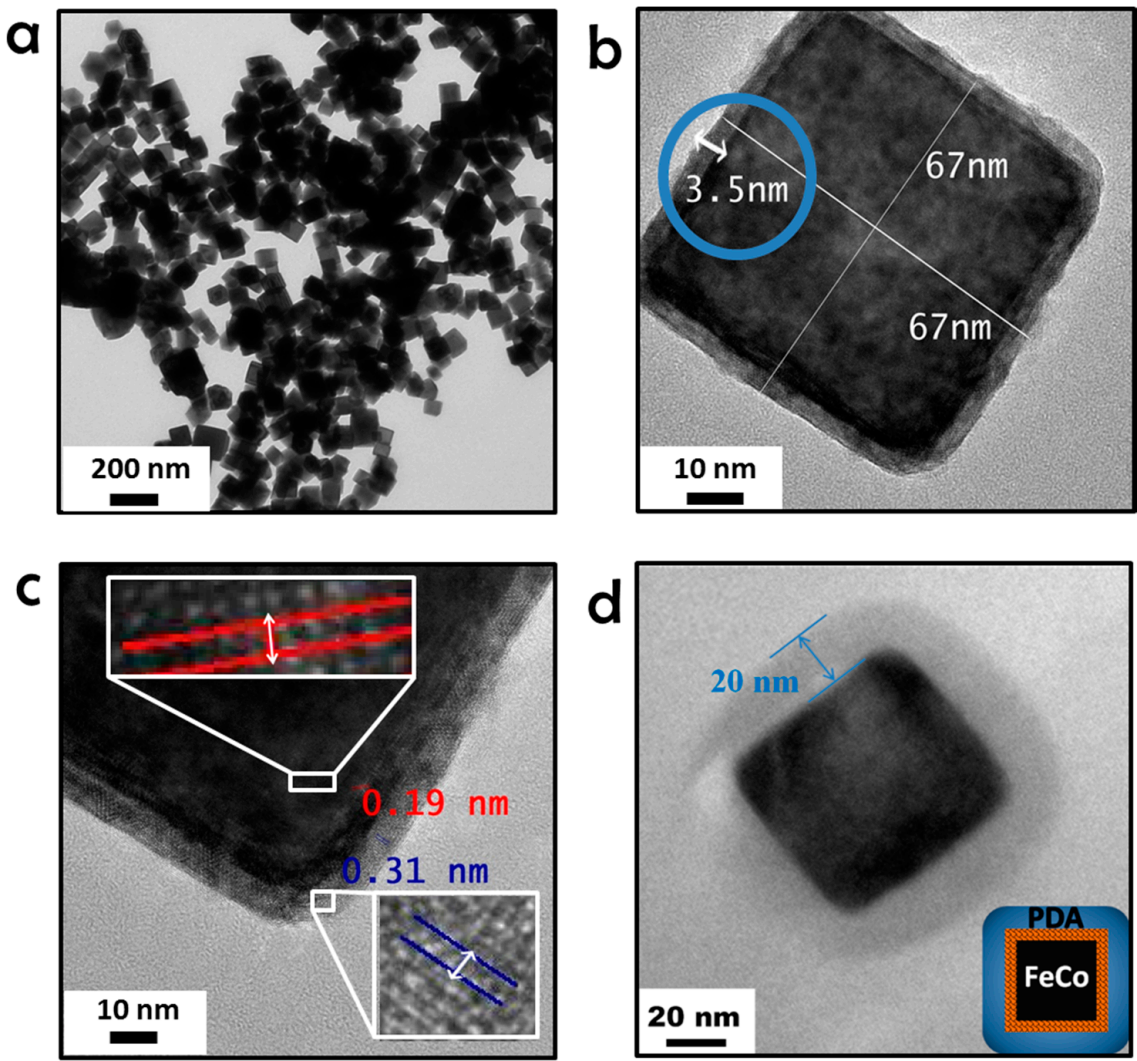
(**a**) TEM images of the as prepared FeCo NCs; (**b**) HR-TEM image of FeCo·(Fe,Co)*_x_*O*_y_* NCs after oxidation in toluene; (**c**) oxide shell is 3.5 nm on HR-TEM image of the FeCo·(Fe,Co)*_x_*O*_y_* NCs with zoomed areas of crystal lattices distances for metal core (red) and oxide shell (blue); (**d**) TEM image of PDFC with 20 nm polydopamine shell.

Single crystalline body centered FeCo cubic nanoparticles (60–70 nm) (Figure 1a) were prepared following reported procedure with slight modifications (Figure S1A) [9]. Briefly, to an aqueous solution of PEG-200, FeSO_4_·7H_2_O, CoSO_4_·7H_2_O and heptane were added consecutively. The solution was sonicated for 80 min. Finally, a solution of NaOH and hydrazine was added to the reaction and the whole mixture was heated at 78 °C until the light pink color changed to dark brown. The nanocubes were separated by centrifugation and washed five times with water and absolute ethanol before drying in a vacuum oven. Oxidation was done in a toluene solution of trimethylamine *N*-oxide to give an air stable structure (Figure 1b) and allow for dopamine polymerization on the surface after adjusting the pH of solution. High resolution TEM (Figure 1c) showed lattice fringes of the FeCo-core (*d* spacing = 1.9 Å) and oxide shell (*d* spacing = 3.1 Å). The elemental composition of nanocrystals was determined by energy dispersive X-ray analysis (EDAX). HR-TEM-EDAX (Figure S2A) pattern demonstrates atomic composition as 68% of Fe and 32% of Co, which is close to the expected 65% and 35% respectively. Furthermore, SEM-EDAX (Figure S2B) pattern clearly exhibits the same atomic ratio of metals in the oxide shell. Powder X-Ray diffraction (XRD) and electron diffraction pattern from single nanoparticle (SAED) (Figure S3) prove the high crystallinity of FeCo nanocubes.

The uniform polydopamine coating of a thickness of 20 nm was verified by TEM (Figure 1d). Further characterization by FTIR showed aliphatic and aromatic C-H stretching modes at 1043 and 974 cm^−1^ (Figure S4). Thermogravimetric analysis (TGA) of PDFCs also validates the presence of an organic shell with significant weight loss at 600 °C according to the first derivative of the thermogravimetric (DTG) curve, leading to a total weight loss of 26% (Figure S4). Magnetization measurements were done by a superconducting quantum interference device (SQUID) and showed that PDFCs had a saturation magnetization (M_s_) of 163 emu/g at 300 K (Figure S5A). Zero-field-cooled (ZFC) (300 to 2 K) and field-cooled (FC) magnetization curves demonstrate typical superparamagnetic behaviour (Figure S5B).

### 2.2. Biocompatibility and Microwave-Induced Chemotoxicity

**Figure 2:**
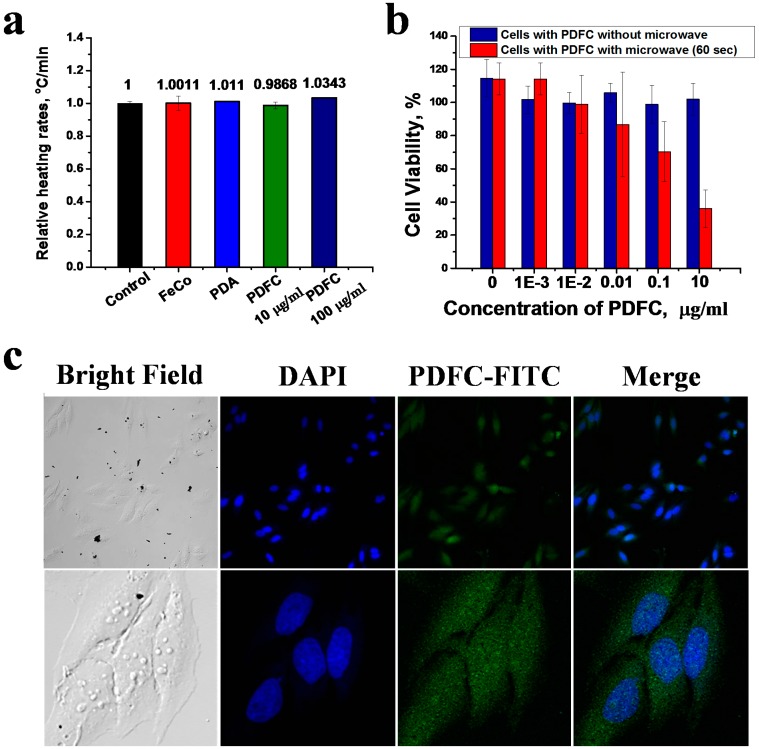
(**a**) Relative heating rates for, FeCo, PDA and PDFC calculated from the slopes of temperature vs time graphs and divided on empty electrolyte heating rate used as control; (**b**) The effect of microwave on cells with internalized PDFC, which indicates obvious reduction in viability compared to controls; (**c**) CLSM imaging of HeLa cells showing successful internalization of PDFC. PDFC was labeled by FITC to track nanoparticles by green fluorescence (excitation and emission wavelengths are 488 and 500–600 nm, respectively).

The biocompatibility of PDFCs was investigated at different concentrations, with no obvious toxicity up to the 100 µg/mL concentration (Figure S6). Two concentrations of PDFCs (10 and 100 µg/mL) were then dispersed separately in aqueous solutions and their relative heating rates were measured in response to microwave irradiation (2.45 GHz frequency, 0.86 W/cm^2^ power, 60 s) (Figure 2a). Interestingly, the thermal increase at both concentrations was identical to that observed with water alone. Incubating HeLa cells with PDFCs at different concentrations in the presence and absence of microwave irradiation showed that with no irradiation HeLa cells had high viability, however, viability started decreasing in the presence of only 0.01 µg/mL of PDFCs, reaching 30% at 10 µg/mL under microwave irradiation (Figure 2b). To verify that toxicity was caused by uptaken PDFCs, their internalization ability with HeLa cells was studied by confocal laser microscopy (CLSM) using dye labeled PDFC (PDFC-FITC). FITC was successfully coupled to the polydopamine shell (Figure S1B) so the nanocubes could be tracked by their green fluorescence. CLSM images showed efficient uptake of most of the PDFC-FITC nanoparticles in 6 h (Figure 2c). Uptake was quantified by inductively coupled plasma optical emission spectrometry (ICP-OES) measurements (Figure S8) of Co and Fe concentrations after the 6 h of incubation with HeLa cells, using of initial dispersions of PDFCs in cell culture medium as controls The uptake was 45.7% for 100 µg/mL, 48.6% for 10 µg/mL and 41.8% for 1 µg/mL based on Fe. Based on Co it was 44.9% for 100 µg/mL, 46% for 10 µg/mL and 35.4% for 1 µg/mL.

### 2.3. Microwave-Induced Toxicity

**Figure 3:**
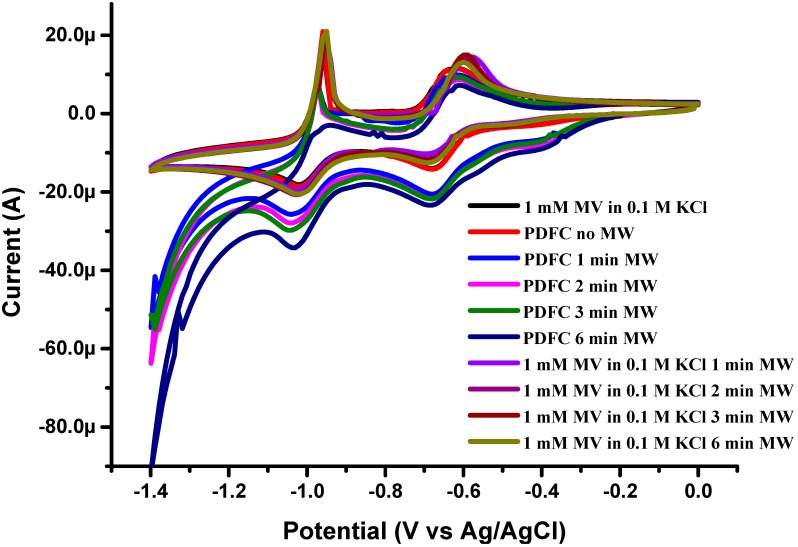
Cyclic voltammetry of 1 mM methyl viologen (MV) in 0.1 M KCl, demonstrating correlation of exposure time and changes in redox states of MV. Scan rate is 50 mV·s^−1^.

Given the lack of differential heating of the nanocube solution, an alternative mechanism to simple hyperthermia is required. We hypothesized that microwave treatment could induce redox processes that would release toxic ions. Evidence for this release is provided by cyclic voltammetry, using a well-known electroactive compound, namely methyl viologen [17]. Methyl viologen hydrochloride hydrate (MV) was dissolved in deionized water containing KCl leading, to the formation of 0.1 mM solution of MV in 0.1 M KCl. The solution was then degassed by purging nitrogen for 2 h. The MV solution showed no change in voltammetric behavior during microwave exposure (Figure 3).

Similar results were observed when PDFCs were dispersed in MV solution without microwave radiation. In contrast, obvious changes were detected in the set of experiments with suspensions of nanocubes exposed to microwave (Figure 3). Moreover, these changes were dependent on irradiation exposure, indicating that the nanocubes either generated interfering species or served directly as redox units for the MV. It is clear that organic species are involved in this toxicity, as only trace amounts of metals were released after the microwave exposure (0.01 ppm for Co and 0.1 ppm for Fe, respectively), around four orders of magnitude less than the established toxic concentrations of these ions [18,19].

## 3. Experimental Section

### 3.1. Materials

PEG 200 and dopamine hydrochloride were obtained from Fluka (Sigma-Aldrich, St. Louis, MO, USA) and Alfa Aesar (A Johnson Matthey Co., Ward Hill, MA, USA), respectively, and were of analytical grade. The 85 wt % hydrazine hydrate and sodium hydroxide pellets were obtained from Sigma-Aldrich and were of reagent grade. The iron sulfate heptahydrate and cobalt sulfate heptahydrate were obtained from Alfa Aesar (A Johnson Matthey Co., Ward Hill, MA, USA) at 99.999% of metal purity, based on metal content. All other chemicals were obtained from Sigma-Aldrich (St. Louis, MO, USA) and were of analytical grade.

### 3.2. Methods

Transition electron microscopy (TEM) images were recorded on Tecnai Bio Tween T12 TEM microscope (FEI Co., Hillsboro, OR, USA), high resolution (HR) TEM and HR-TEM-energy dispersive X-ray spectra (EDX) analysis were carried out on Titan CT microscope (FEI Co.). The Scanning electron microscopy (SEM)-EDX analysis was carried out on a Quanta 200 FEG microscope (FEI Co.). X-ray powder diffraction (XRD) was carried out on a Bruker D8 Advance X-ray diffractometer with Cu Kα (λ = 1.5406 Å) in the 2θ ranges. Magnetization was measured on a Magnetic Property Measurement System—MPMS^®^ SQUID VSM (Quantum Design International, San Diego, CA, USA), UV-VIS-NIR spectra was recorded on a Varian 5000 spectrophotometer. FT-IR spectra were recorded on a Thermo Nicolet iS10 spectrophotometer (Thermo Fisher Scientific, Waltham, MA, USA). Thermal gravimetric analyses (TGA) were done on a TG 209 F1 machine (NETZCHS, Selb, Germany) Confocal microscopy images were recorded on a Zeiss 7MP multiphoton microscope (ZEISS, Oberkochen, Germany).

### 3.3. Synthesis of FeCo Nanocubes

The typical procedure (Figure S1A) of Wei *et al* [9] was used with minor modifications, as described for synthesis of the FeCo nanocubes with atomic ratio of Fe/Co to 65/35; the ratio at which alloy has the highest saturation magnetization is well known. The first solution A was prepared: 6.88 g of PEG-200 (34.4 mmol) were dissolved in 50 mL of deionized and degassed water (DDW) (3 cycles of freezing under N_2_ atmosphere/warming up under vacuum) following the addition of 720 mg of FeSO_4_·7H_2_O (2.589 mmol) and 392 mg of CoSO_4_·7H_2_O (1.394 mmol). Solution A was stirred under an N_2_ atmosphere until complete dissolution of salts. Then, 0.8 mL of heptane added via syringe and the emulsion was placed in a bath sonicator for 80 min. Solution B was prepared in another flask: 2.45 g of sodium hydroxide (61.2 mmol) was dissolved into 20 mL of hydrazine hydrate 85% water solution (0.34 mol) and purged with N_2_ for 15 min, followed by the addition of 0.1 mL of heptane and sonication for 1 h. Solution A was heated with stirring to 78 °C and Solution B was added within 1 min under an N_2_ atmosphere. The color of solution changed from light pink to green first, and then, within seconds, darkened to brown. After 1.5 h of stirring, the obtained nanoparticles were separated by a rare earth magnet and washed 5 times with DDW until the pH of the washings became neutral, and, finally, washed twice with absolute ethanol and dried under vacuum for 4 h at 40 °C. TEM images show the alignment of nanocubes in long chains and bigger agglomerates because of the remaining magnetization after separation with the magnet. For all next steps, including controlled oxidation, polymer coating, and characterization experiments, mechanical stirring and separation with a centrifuge at 4000 rpm for 5 min were used to obtain non-magnetic material for better dispersion. It is crucial to keep this material far from magnetic fields to prevent their rapid agglomeration prior to use in biological experiments. Nanoparticles were dispersed with sonication in the solution of 30 mg of trimethylamine *N*-oxide in toluene (0.4 mmol), purged previously with N_2_ for 2 h, and stirred with a mechanical stirrer for 2 h at 70 °C. Nanocubes were then isolated by centrifugation at 4000 rpm for 5 min, washed with absolute ethanol 5 times and dried for 4 h under vacuum.

### 3.4. Polydopamine Coating of FeCo Nanocubes—PDFC

The procedure of coating was done through the polymerization of dopamine where 60 mg of FeCo nanoparticles were dispersed in 120 mL of deionized water containing 120 mg of dopamine hydrochloride with intensive sonication. Thirty minutes of sonication with shaking dispersion showed no visible aggregates (dark grey but transparent under the light). The pH of the solution was adjusted with potassium hydroxide (0.01 M solution) to 8.5 and mechanically stirred under ambient conditions. After 12 h of stirring, pH dropped to 6.4, which was again adjusted to 8.5 and stirring continued for another 12 h. Obtained nanoparticles were separated by 10 min of centrifugation at 4000 rpm and washed 5 times with deionized (DI) water, two times with ethanol, and dried in a freeze drier for 12 h prior to characterization. The remaining solution containing polydopamine without FeCo nanoparticles was transferred into a dialysis membrane with an MW cutoff of 1000 and dialized against DI water for 2 days to remove low molecular weight by-products and dopamine. After purification, water was removed in a freeze drier and dark brown-to-black sticky polymer was used to compare with PDFC nanoparticles. A total of 26% *w*/*w* PDA containing composite was obtained.

### 3.5. FITC Dye Attachment—PDFC-FITC

One milligram of PDFC was dissolved in degassed and deionized water containing 1 mg of FITC isocyanate by intense sonication for 15 min in an ultrasonic bath, then, one drop of 10% sodium bicarbonate solution was added and left to shake intensively overnight. The nanoparticles were separated by centrifugation for (4000 rpm) 3 min and washed with water 6 times to remove excess FITC. PDFC-FITC nanoparticles were dried under vacuum for 2 days.

### 3.6. Magnetic Characterization

Different conditions were chosen—two temperatures of 2 and 300 K for the response of magnetization change at Field Cooling (FC) and Zero Field Cooling (ZFC). Saturation magnetization gave 163.2 emu/g (Figure S5A) at 300 K. Considering the fact that the magnetic core of nanocomposite is 74% of the total weight (TGA for this sample showed 26% of polydopamine content PDA) recalculation of the magnetization of the core gives 226.6 emu/g. Low coercivity value could be explained as good dispersibility of the sample—in the case of more interaction between two nanocubes (contact) the higher the coercivity will be. This experiment showed that magnetic cores are separated from each other by a non-magnetic material, polydopamine.

### 3.7. Microwave Experiments

Heating rates were measured via parallel registration of temperature rises using two optical fiber temperature probes, placed deep in vials containing electrolyte solution with dispersions of polydopamine, as-synthesized FeCo nanocubes and PDFC nanocomposite. During measurement, power distribution was homogeneous while two identical electrolyte samples with the same masses in identical vials were tested (Figure S7A). Although, within one experiment, the power of microwave was distributed uniformly, after switching on and off, the power was slightly different. Thus, for accurate control within every measurement, a control sample of 0.1 M KCl electrolyte was used as standard and results of all heating rates were normalized according to this standard (Figure S7B).

### 3.8. Cell Culture Studies

HeLa cells were grown in 96 well plates at a density 5000 cells per well in EMEM cell culture media containing 10% of FBS and 0.1% penicillin–streptomycin, at 37 °C in a humidified 5% CO_2_ atmosphere. For the cell viability test (Figure S6) HeLa cells were incubated with PDFCs at the concentrations: 100, 10, 1, 0.1, 0.01, 0.001, 10^−4^, 10^−5^ and 10^−6^ µg/mL, and incubated for 12 h prior to addition of WST-8 dye—(2-(2-methoxy-4-nitrophenyl)-3-(4-nitrophenyl)-5-(2,4-disulfophenyl)-2H-tetrazolium. monosodium salt)—and then incubated for an additional 3 h according to the CCK-8 standard protocol for colorimetric measurement of cell viability. In the case of the cell internalization study of PDFCs by ICP-OES, cells were incubated with 10, 1, 0.1 and 0.01 µg/mL of nanoparticles for 6 h. Consequently media containing non-internalized PDFCs was removed and cells were washed with fresh media, which was combined with the first portion. Then, the cells, containing the internalized PDFCs, were harvested by trypsination and the amounts of Co and Fe were analyzed by ICP-OES along with the supernatant, containing non-internalized PDFCs.

### 3.9. Confocal Laser Scanning Microscopy Study

HeLa cells were seeded on glass cover slides, and cultured in EMEM medium containing 10% FBS and 0.1% penicillin–streptomycin at 37 °C in a humidified 5% CO_2_ atmosphere. After cell attachment, the media was replaced by fresh media containing 10 µg/mL of PDFC-FITC, followed by incubation for 12 h. Cells on cover slides were washed twice with DPBS, then fixed with 4% paraformaldehyde for 1 h and washed 3 times with DPBS. Then, nuclei were stained with DAPI for 3 min and then washed 3 times with DPBS. Finally, cells were observed with confocal laser scanning microscopy (CLSM, Zeiss LSM 710 upright confocal microscope, (Zeiss, Oberkochen, Germany), with excitation and emission wavelengths at 488 and 500–600 nm, respectively).

## 4. Conclusions

In summary, we have showed that polydopamine coated iron-cobalt single crystalline nanoparticles at very low concentrations led to non-thermal cancer cell death under 2.45 GHz microwave irradiation at low power. Specific absorption rate measurements demonstrated that the mechanism of death was not related to the rise of temperature (hyperthermia). We hypothesize that microwave irradiation is inducing a degradation of the polydopamine monolayer, inducing toxicity. We are further exploring the mechanism and application of this novel process.

## Supplementary Materials

Supplementary materials can be found at http://www.mdpi.com/1422-0067/16/08/18283/s1.
