# Supplementary material for: Microwave-Induced Chemotoxicity of Polydopamine-Coated Magnetic Nanocubes
Source: Int J Mol Sci. 2015 Aug 6;16(8):18283–92. doi: 10.3390/ijms160818283 (PMC4581245; doi:10.3390/ijms160818283)
Supplement: Supplementary file 1 [file ijms-16-18283-s001.pdf]

Supplementary Information

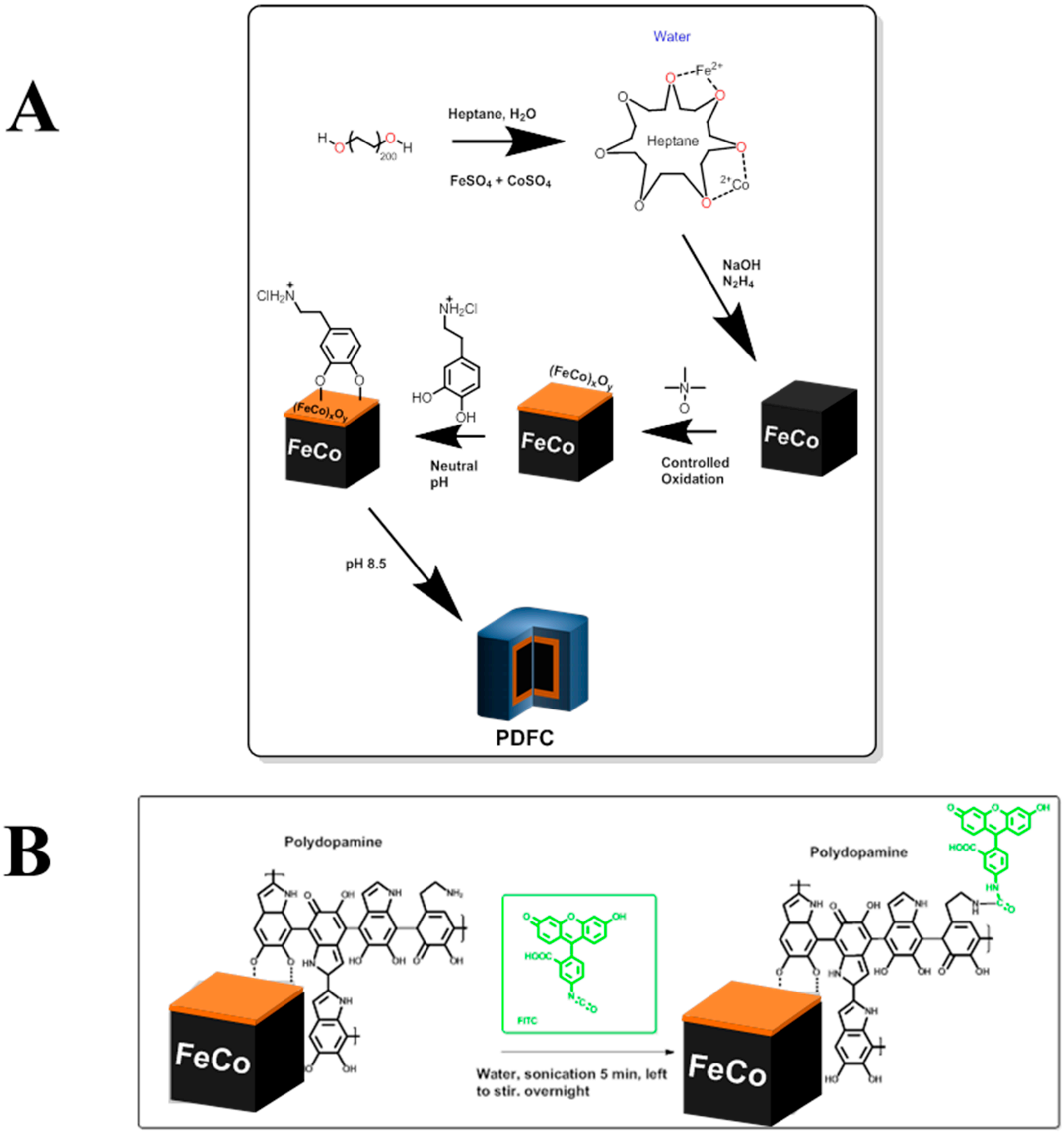

Figure S1. (**A**) Synthetic scheme of PDFC and (**B**) PDFC-FITC.

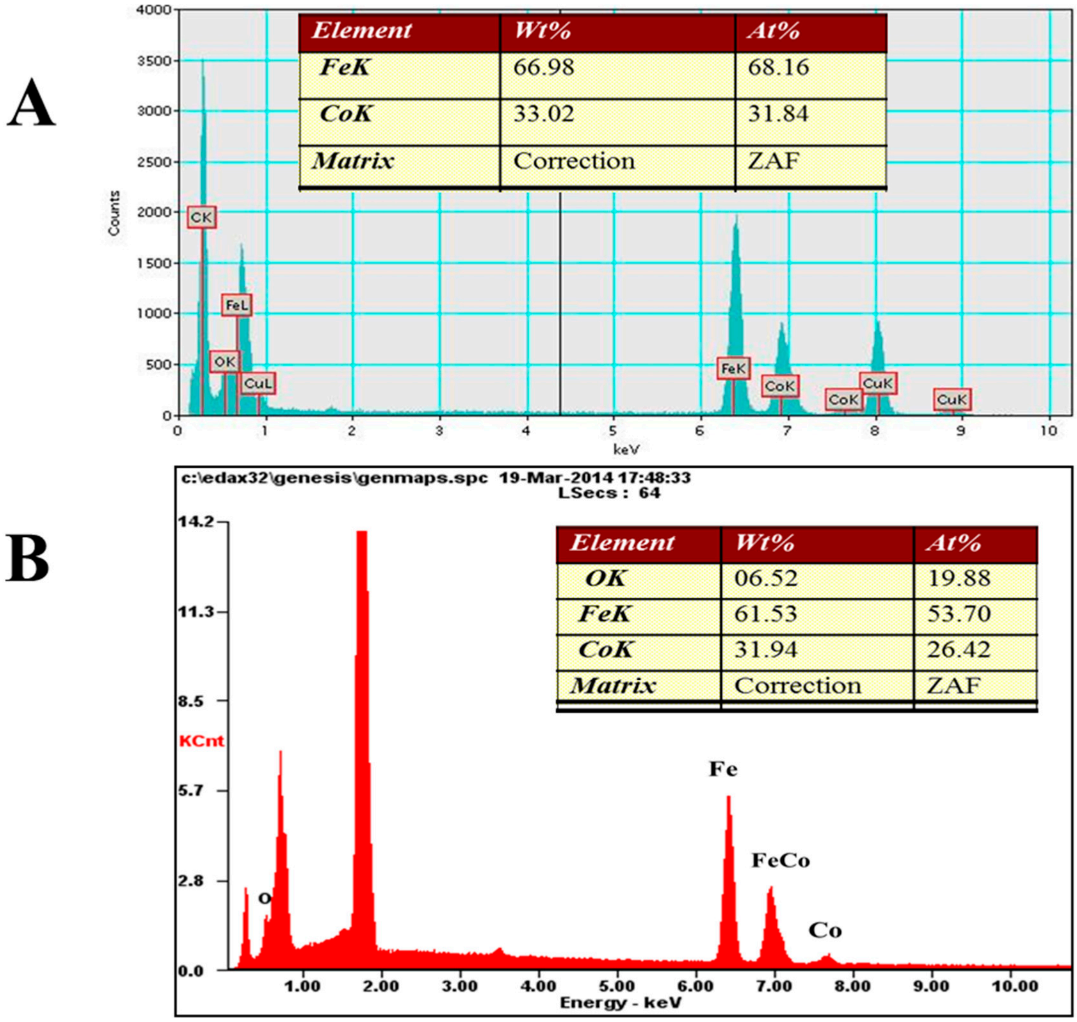

Figure S2. (**A**) TEM-EDAX and (**B**) SEM-EDAX for FeCo nanocubes.

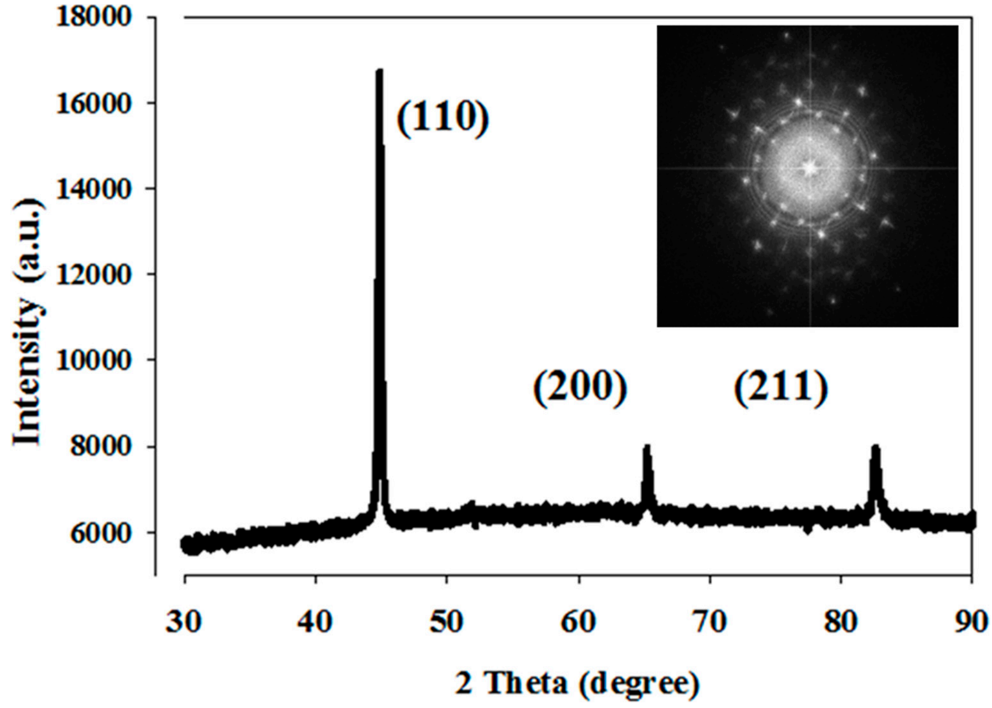

Figure S3. Powder XRD with SAED included for FeCo nanocubes.

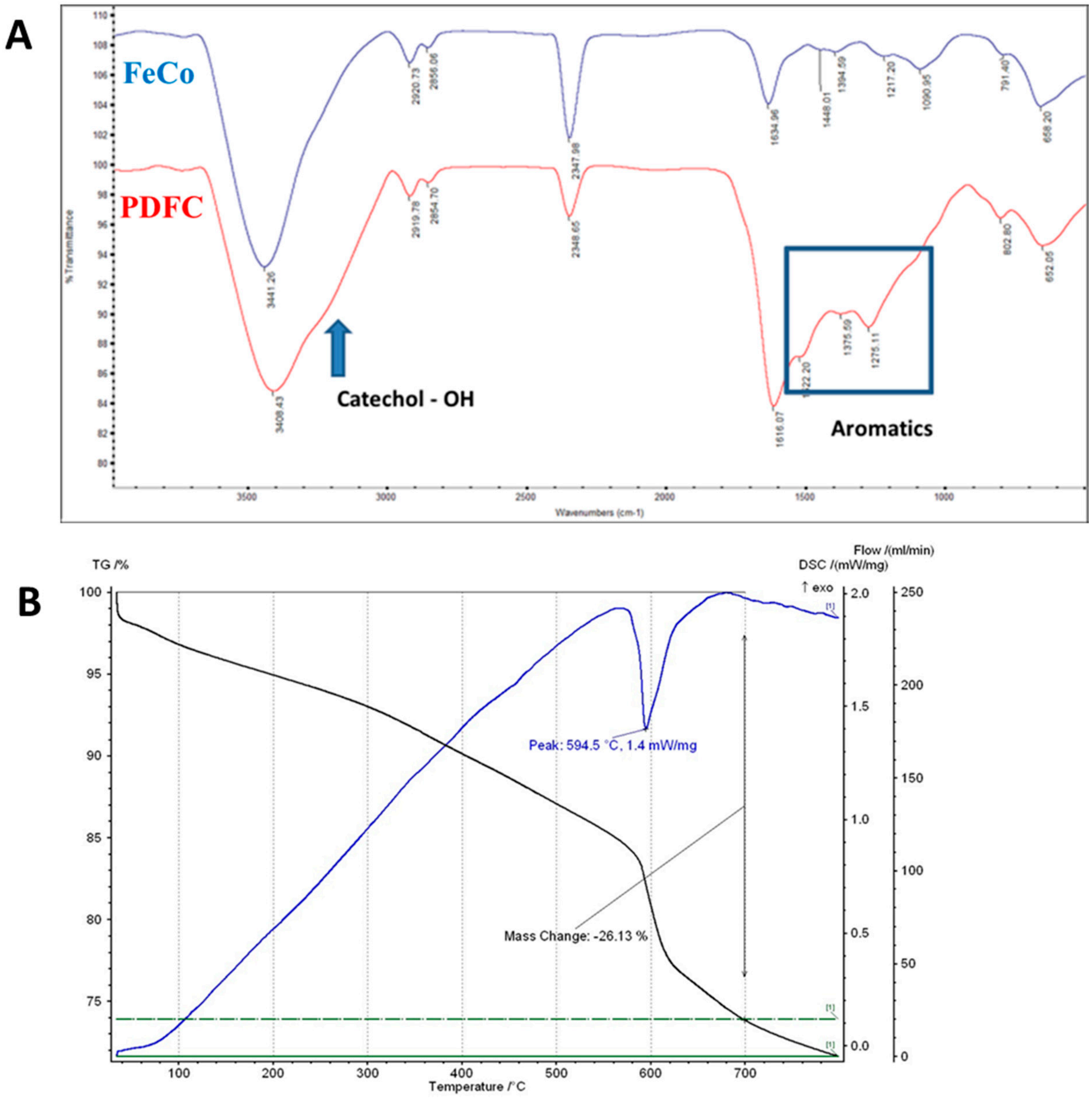

Figure S4. (**A**) FTIR spectra for FeCo and PDFC; (**B**) TGA and DTG curves for PDFC.

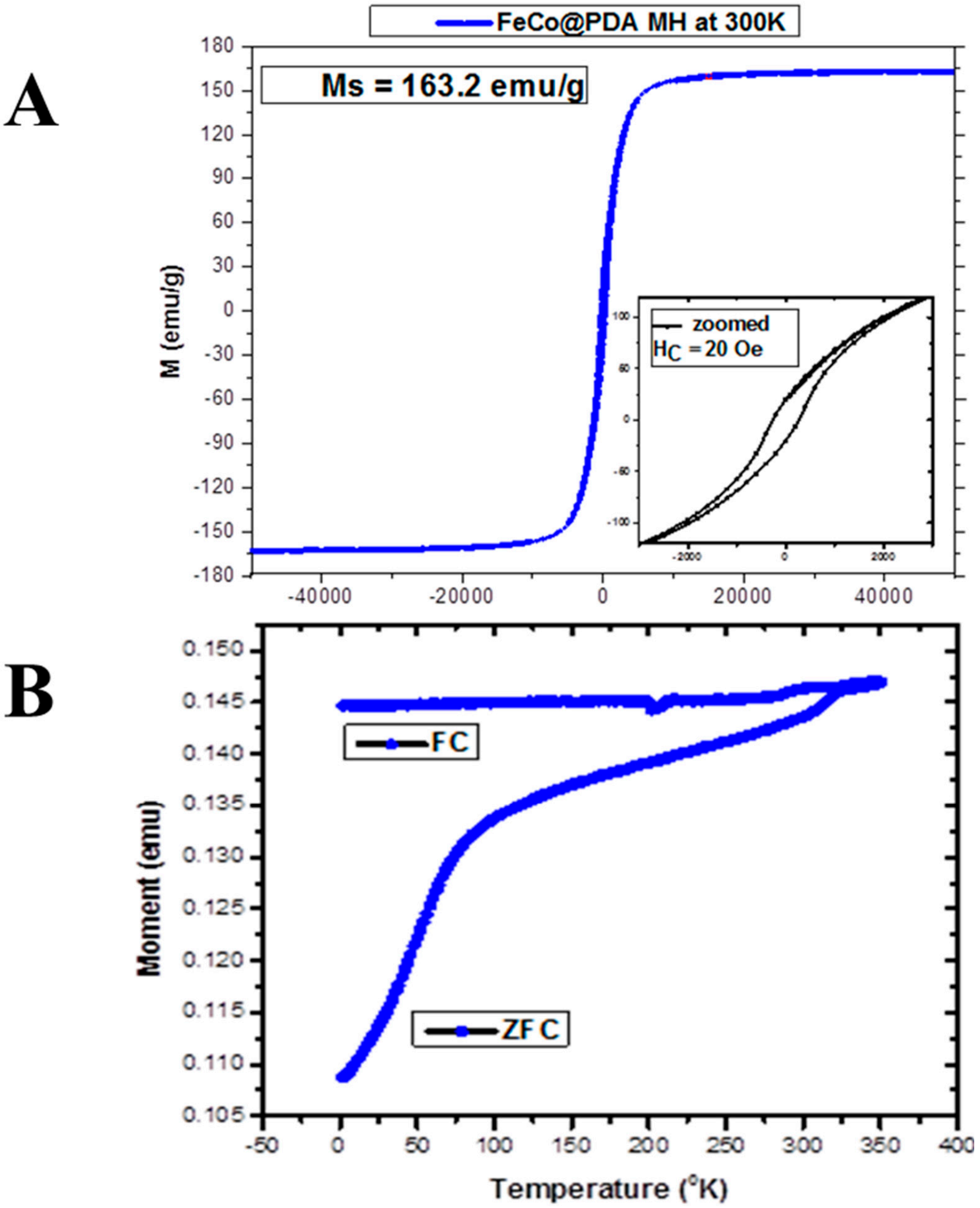

Figure S5. (**A**) MH SQUID magnetization curve and (**B**) FC, ZFC curves for PDFC.

A

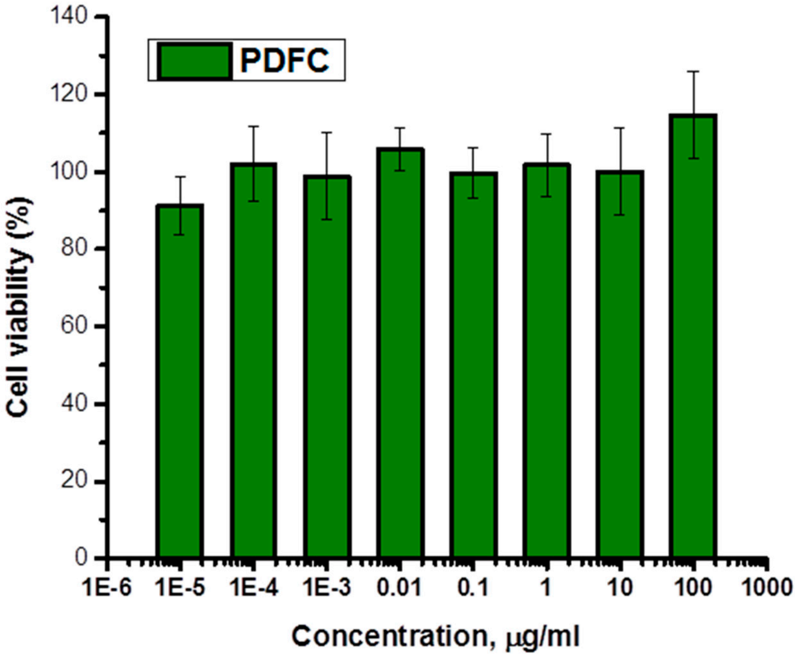

B

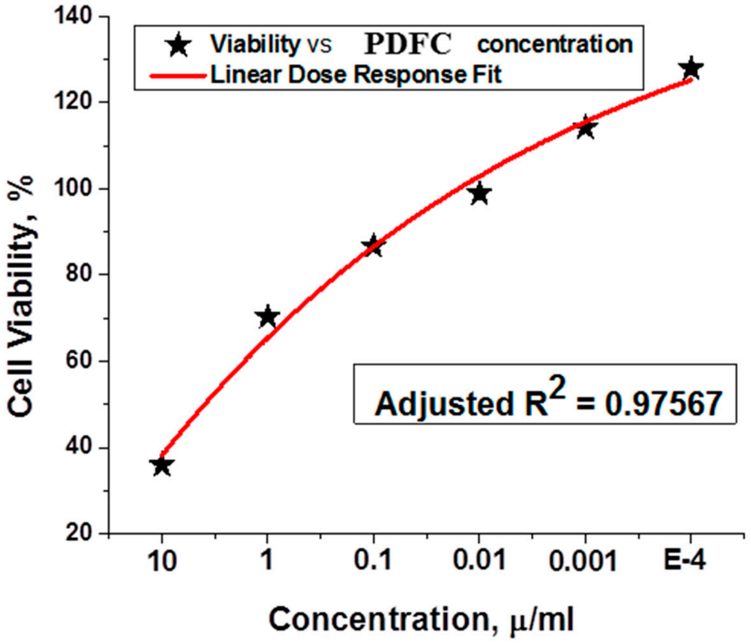

Figure S6. (**A**) Biocompatibility test for PDFC and (**B**) Linear dose response for the MW + PDFC.

A

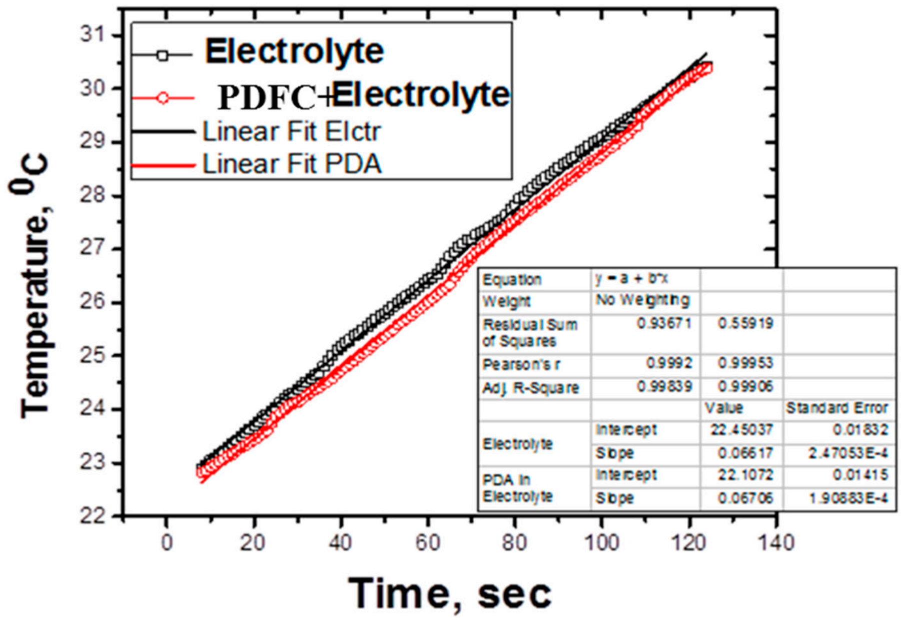

B

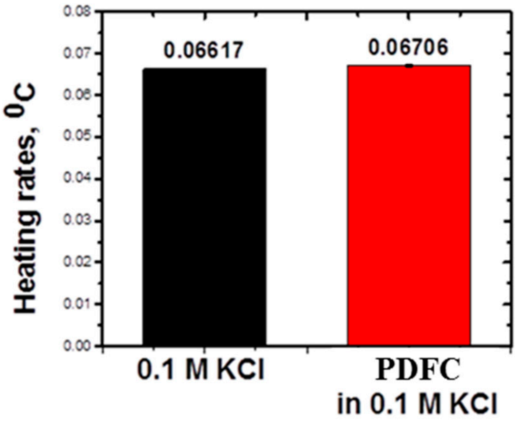

Figure S7. Typical graphs for heating rates calculations. (**A**) the temperature *vs.* time plot for 0.1 M KCl electrolyte and the same electrolyte containing 100 mg/mL PDFC; (**B**) Heating rates comparison, calculated from the slopes of linear fits on graph (**A**).

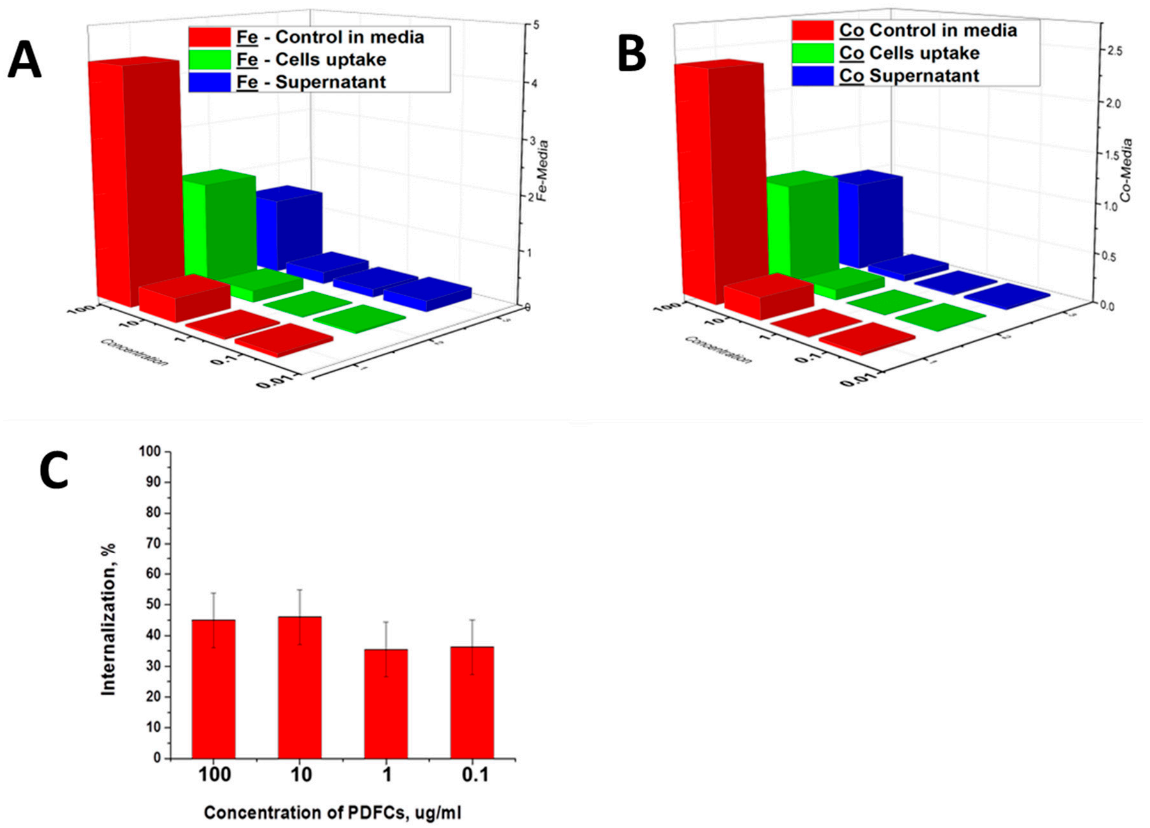

Figure S8. Internalization of PDFCs measured by ICP-OES: The red bar represents the amounts of metals (Fe (**A**) and Co (**B**)) in ppm for the initial dispersion of PDFCs in cell culture media used to feed the cells. The green bar represents the amount of metal in cells after the 6 h of incubation with PDFCs following the removal of culture media, washing with PBS buffer and harvesting by trypsin. Respectively, the blue bar shows the amount of metals in the washings. The graph (**C**) shows relative percentage of uptake (based on Co) for different concentrations compare with initial dispersions of PDFCs.
